# Identification and characterization of cytosolic malate dehydrogenase from the liver fluke *Fasciola gigantica*

**DOI:** 10.1038/s41598-020-70202-y

**Published:** 2020-08-07

**Authors:** Purna Bahadur Chetri, Rohit Shukla, Timir Tripathi

**Affiliations:** 1 Molecular and Structural Biophysics Laboratory, Department of Biochemistry, North-Eastern Hill University, Shillong, 793022 India; 2 Present Address: Department of Biotechnology and Bioinformatics, Jaypee University of Information Technology, Waknaghat, Solan, 173234 India

**Keywords:** Biophysical chemistry, Computational chemistry, Protein structure predictions

## Abstract

The liver fluke zoonoses, *Fasciola* spp. are parasitic helminths infecting humans and animals globally. Recent sequencing of the genome of *Fasciola gigantica* has provided a basis to understand the biochemistry of this parasite. Here, we identified the cytosolic malate dehydrogenase in *F. gigantica* (FgMDH) and characterized the enzyme biochemically and structurally. *F. gigantica* encodes a single cytosolic MDH, a key enzyme of the citric acid cycle. It catalyzes the reversible oxidation of malate to oxaloacetate using NAD^+^. The *Fgmdh* gene was amplified and cloned for expression of the recombinant protein. The purified protein showed a molecular weight of ~ 36 kDa that existed in a dimeric form in solution. The recombinant enzyme was catalytically active as it catalyzed both forward and reverse reactions efficiently. The kinetic parameters were determined for both directions. The structure of FgMDH and human MDH were modeled and validated. The superimposition of both the model structures showed overall structural similarity in the active site loop region, however, the conformation of the residues was different. Molecular docking elucidated the binding sites and affinities of the substrates and cofactors to the enzyme. Simulation of molecular dynamics and principal component analysis indicated the stability of the systems and collective motions, respectively. Understanding the structural and functional properties of MDH is important to better understand the roles of this enzyme in the biochemistry of the parasite.

## Introduction

Two parasitic liver flukes, *Fasciola gigantica* and *Fasciola hepatica* are responsible for fascioliasis—a severe zoonotic disease that is financially detrimental to the global livestock industry^1^. WHO classifies fascioliasis as a neglected tropical disease of economic importance^2^. It is a major foodborne disease caused by the consumption of food/water contaminated with the parasites in their larval stage. It is primarily a veterinary disease that infects a variety of livestock, especially goat, sheep, and cattle^3–5^; globally, over 600 million animals are affected by these flukes, resulting in loss of > US$3 billion p.a. *F. gigantica* is prevalent in tropical regions, including Africa and South and Southeast Asia, while *F. hepatica* is mostly found in temperate areas, including Europe, North and South America, and Australia^6^. *F. gigantica* infects nearly 25–100% cattle annually in Africa, the Middle East, Southeast Asia, and other temperate countries^7^. The global prevalence of fascioliasis is increasing in humans. As reported by WHO, nearly 180 million people are at risk, while 2.4–17 million people are infected globally^8^. Human fascioliasis is primarily defined to Africa, Europe, the Middle East (including Egypt), Southeast Asia, and Latin America with the highest prevalence in Bolivian Plateau^9,10^. The common symptoms of fascioliasis are enlarged liver and spleen, arthralgias, facial swelling, fever, abdominal pain, eosinophilia, and eggs in stool^11^. The only drug of choice recommended by WHO is triclabendazole (TCBZ)^12^. However, there have been several reports of the appearance of resistance against TCBZ globally^13,14^.

Targeting metabolic enzymes for structure-based drug discovery is an effective strategy for the treatment and management of infectious diseases^15^. Malate dehydrogenase (MDH; EC 1.1.1.37) is a ubiquitous enzyme present in almost all eukaryotic organisms. It is an essential tricarboxylic acid (TCA) cycle enzyme that is involved in energy metabolism in eukaryotic cells. It catalyzes the interconversion of malate to oxaloacetate with subsequent production of NAD(H); thus, it is NAD-dependent dehydrogenase^16^. MDH exists in two isoforms, namely mitochondrial MDH (mMDH), which is involved in TCA cycle, and cytosolic MDH (cMDH), which is involved in malate–aspartate shuttle wherein it transfers the reducing equivalents from the cytosol to mitochondria in the form of malate/oxaloacetate instead of (NAD/NADH)^17–19^. cMDH is also involved in several other metabolic pathways, such as gluconeogenesis^20^, glyoxylate cycle^21^, glyoxylate degradation^22^, and mixed acid fermentation^23^. The 3D structures and active sites are conserved between mMDH and cMDH, although these isoenzymes are only slightly related at the level of amino acid sequence. Several crystal structures of MDH are available in both apo- and substrate analogs/inhibitor-bound forms^24^. Structurally, MDH belongs to lactate dehydrogenase (LDH)/MDH superfamily and are classified into three groups: LDH (tetrameric enzymes), LDH-like MDH (tetrameric enzymes), and MDH (dimeric enzymes)^25^. Each subunit is made up of two structurally and functionally distinct domains, i.e., an N-terminal coenzyme with NAD^+^ binding domain consisting of a parallel β-sheet structure (a Rossman fold). The central NAD^+^ binding site is composed of four β-sheets and one α-helix. The C-terminal domain contains the substrate-binding site and residues necessary for catalysis. The active site is located in a cleft between the two domains^24^. The dimer interface consists of interacting α-helices. The active site of MDH consists of a hydrophobic cavity with specific binding sites for the substrate and coenzyme NAD^+^. Upon the formation of the enzyme–coenzyme–substrate ternary complex, a conformational change occurs during which the external loop flips to block the active site from the solvent. Other residues of the active site are then brought closer to the substrate to aid the conversion of the substrate to its product^18,26^.

Expression of MDH protects *Escherichia coli* against oxidative stress, suggesting the physiological function of MDH in bacteria^27,28^. It has been proposed that both cMDH and mMDH are involved in regulating energy metabolism in parasitic helminths^29,30^. cMDH is also involved in the anaerobic metabolism upon penetration of the flukes in the host bile ducts^31^. During anaerobic metabolism, phosphoenolpyruvate (PEP) is converted to oxaloacetate via phosphoenolpyruvate carboxykinase (PEPCK), which is subsequently reduced by cMDH, reoxidizing the glycolytic NADH. This malate is transported to the mitochondria, where it is dismutated. Some malate is oxidized to acetate, while some are reduced to succinate and then metabolized to propionate. Thus, malate provides a source of electrons as well as a sink for these electrons^32^. The glycolytic enzymes, including MDH and phosphoglycerate kinase, have been shown to be critical for the survival of trematodes, and consequently, they have been targeted for the development of vaccines and screening of drugs^33,34^. However, to our knowledge, no studies have identified and studied the MDH from *F. gigantica*. Herein, we aimed to identify cytosolic MDH from *F. gigantica* and characterized its biochemical and structural properties.

## Results and discussion

### Multiple sequence alignment and phylogenetic analysis

**Figure 1:**
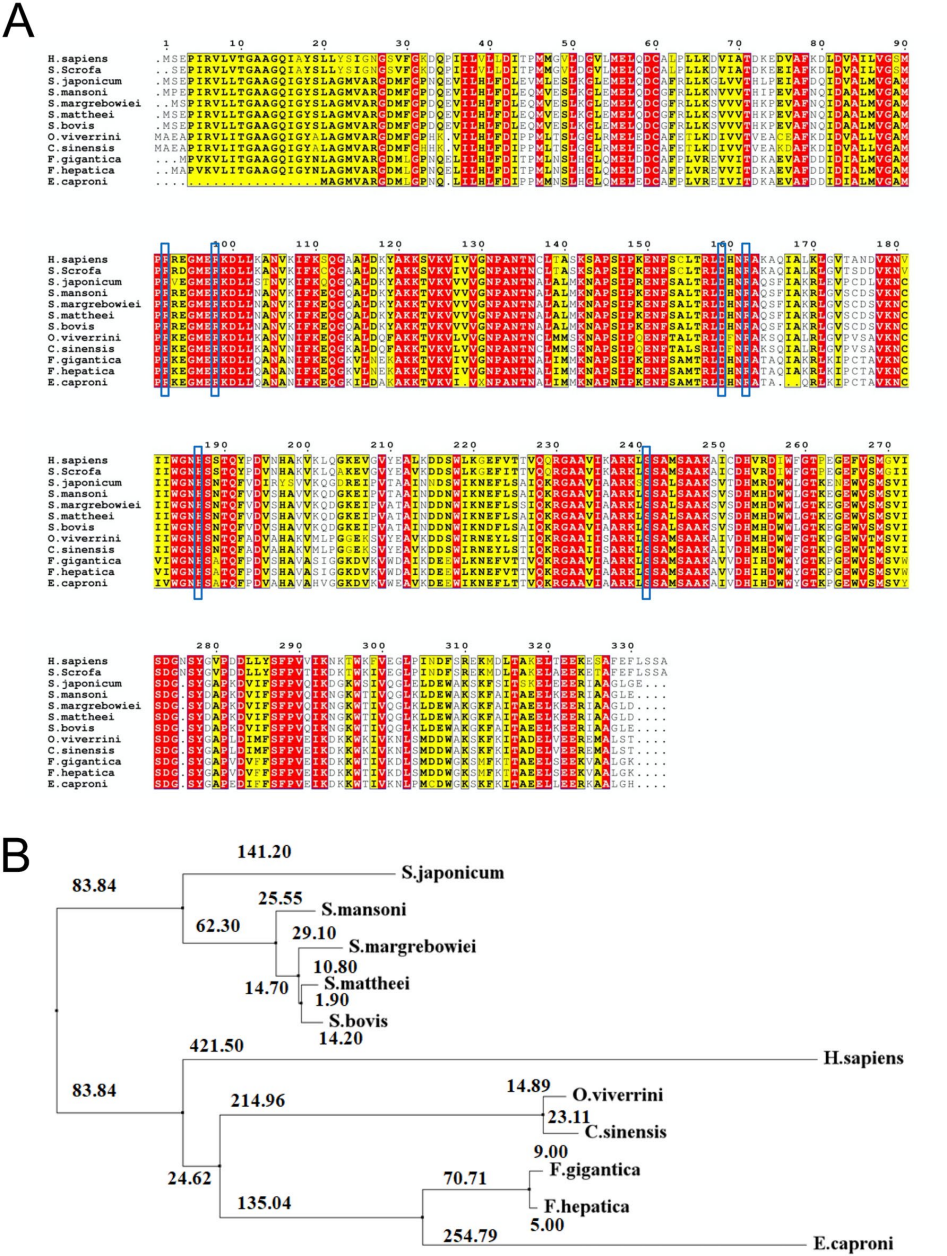
Evolutionary sequence analysis. (**A**) Multiple sequence alignment of FgMDH with MDH from other organisms (*Fasciola hepatica* (THD24885.1)*, Echinostoma caproni* (VDP72865.1), *Opisthorchis viverrini* (OON21665.1), *Clonorchis sinensis* (AAT46071.1), *Schistosoma margrebowiei* (VDO56091.1), *Schistosoma matthaei* (VDP64368.1), *Schistosoma bovis* (RTG88342.1), *Schistosoma mansoni* (XP_018647879.1), *Schistosoma japonicum* (CAX722031.1), and *Homo sapiens* (NP_005908.1). The alignment was generated by ClustalW algorithm. The red boxes show identical amino acids; yellow boxes show similar amino acids, while the amino acids with different properties have no boxes. The blue box indicates the active site residues. (**B**) Phylogenetic relation of the FgMDH. The Neighbour Joining method was used to construct the phylogenetic tree by comparing amino acid sequences by using MEGA V10.0 software^45^. The values represent the evolutionary distance among different species.

The closely related homologs of FgMDH were identified by using BLAST and HMMscan and utilized to ascertain the domains of FgMDH. The result showed that the FgMDH sequence is phylogenetically related to several other cytosolic MDHs. Structurally, the protein encoded two domains, namely N-terminal NAD^+^ binding domain (residues 3–150) and C-terminal binding domain (residues 154–321). The MDH sequences of various organisms were retrieved from NCBI (*Fasciola hepatica, Echinostoma caproni, Opisthorchis viverrini, Clonorchis sinensis, Schistosoma margrebowiei*, *Schistosoma matthaei*, *Schistosoma bovis*, *Schistosoma mansoni*, *Schistosoma japonicum*, and *Homo sapiens*) for phylogenetic analysis. The FgMDH showed 100% sequence identity with MDH of *F. hepatica*, whereas it also showed high sequence similarity with related organisms, such as the MDH of *E. carponi* (88.3%), *O. viverrini* (71.60%), *C. sinensis* (70.99%), *S. margrebowiei* (71.21%), *S. matthaei* (71.21%), *S. bovis* (70.99), *S. mansoni* (71.21%), *S. japonicum* (68.42%), and *H. sapiens* (60.62%) (Fig. 1A). FgMDH showed several conserved residues and motifs that are important for the catalytic mechanism. Sequence motifs, including ^90^RKEGMERKDLL^100^ (active site), ^184^WGNHS^189^, and ^245^SAAK^248^ that are important for the binding of substrate and cofactor are found to be conserved^35^. Residues Arg91, Arg97, and Arg161 (as per *Sus scrofa* numbering) are part of the substrate-binding site and were found to be conserved in all MDHs^35^, including FgMDH (as residues Arg90, Arg96, and Arg160) (Fig. 1A). Residues, such as Gly13, Ile15, Asp41, and Ser240 (*Sus scrofa* numbering) that correspond to Gly13, Ile14, Asp40, and Ser239 in FgMDH are important for cofactor binding and were also conserved. Phylogenetic analysis also presented supporting results. The 100% sequence identity of FgMDH conferred them to the same node. The phylogenetic tree revealed that there were two main clades from the ancestor. In one clade, all the flukes from the *Schistosoma* family were present, while all other flukes, including FgMDH, share the common ancestor in the other clade. The *H. sapiens* MDH also shares the clade with FgMDH; however, it is phylogenetically distant, indicating a substantial evolutionary gap between their evolution (Fig. 1B).

### Cloning, overexpression, and purification of recombinant FgMDH

**Figure 2:**
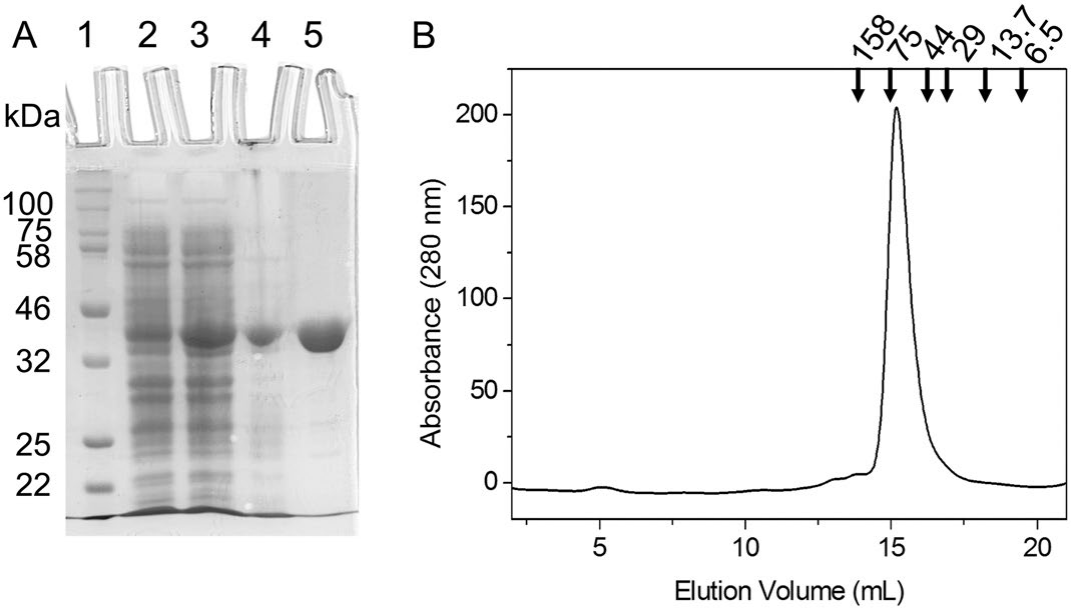
Overexpression, purification, and oligomeric status of recombinant FgMDH. (**A**) SDS-PAGE gel showing the overexpressed and purified protein. Lanes 1–3 represent molecular weight markers, uninduced cell lysate, induced cell lysate, and lane 4–5 represent purified FgMDH, respectively. (**B**) GFC profile showing the oligomeric status and molecular mass of FgMDH. The column calibration was performed with the gel filtration calibration kit containing aldolase (158 kDa), conalbumin (75 kDa), ovalbumin (44 kDa), carbonic anhydrase (29 kDa), ribonuclease (13.7 kDa), and aprotinin (6.5 kDa).

The *mdh* of *F. gigantica* was PCR-amplified using cDNA as a template and a set of primers as described in the methods section. The PCR product corresponded with the expected size (981 bp) of *Fgmdh* (Supplementary Fig. 1). The PCR product was ligated downstream T7 promoter between the Bam HI and Hind III restriction sites of expression vector pET28a( +). The successful ligation was confirmed by PCR using *mdh-*specific primers and restriction digestion of the recombinant plasmid with Bam HI and Hind III (Supplementary Fig. 1). The sequence of cloned *mdh* was confirmed by forward and reverse DNA sequencing. The results showed a 100% identity with the *mdh* nucleotide sequence present in the complete genome sequence of *F. gigantica*^36^. *E. coli* cells harboring the clone were grown in LB media containing kanamycin. The expressed FgMDH was a His-tagged protein that was purified using affinity chromatography. The production of purified recombinant FgMDH was in the range of 20 mg/L. The purified protein was homogenous, as indicated by a single protein band on SDS-PAGE of the molecular mass of nearly ~ 36 kDa (Fig. 2A).

### Determination of molecular weight and oligomeric structure

The molecular mass of the purified FgMDH was determined under non-dissociating conditions by gel filtration chromatography (GFC). The chromatogram of the recombinant protein showed a single peak with a retention volume of about 15.3 mL (Fig. 2B). According to molecular weight standards, this corresponds to a molecular mass of ~ 72 kDa. The result suggests that under the native state the protein exists in a dimeric form in the solution. The existence of dimeric FgMDH is consistent with other MDHs from yeasts, bacteria, and higher eukaryotes^16,37^.

### Enzyme activity and kinetic analysis

**Figure 3:**
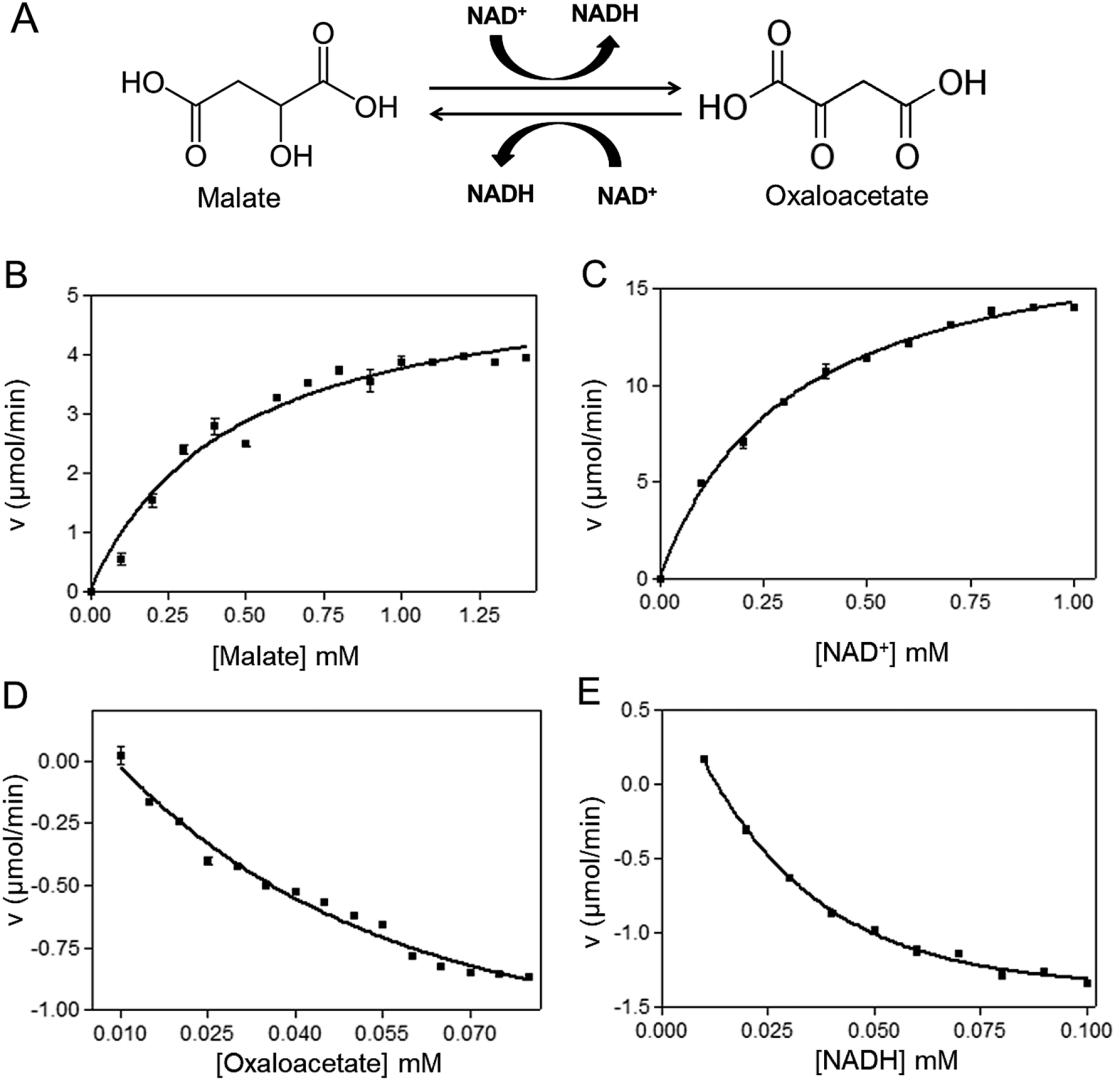
Enzymatic reactions and Michaelis–Menten kinetic plots. (**A**) Schematic diagram of the forward and reverse reactions catalyzed by FgMDH. (**B**–**E**) Michaelis–Menten Kinetic plots for (**B**) malate, (**C**) NAD^+^, (**D**) oxaloacetate, and (**E**) NADH. Enzyme activities were taken in both forward and reverse directions, keeping one substrate/cofactor constant while varying the other.

The recombinant FgMDH was enzymatically active in the solution. It catalyzed both forward (conversion of oxaloacetate to malate) and reverse reactions (conversion of malate to oxaloacetate) under normal conditions. Initial velocity studies were used to determine kinetic constants for substrates and cofactors in the forward and reverse directions. All the kinetic analyses were performed at 25 °C, and the data were calculated using the Michaelis–Menten plot. Table 1 shows the kinetic parameter of recombinant FgMDH. The *K*_m_ value of FgMDH was compared with other related organisms^38–40^, which is shown in Table 2. FgMDH did not show any activity with NADP(H) when used in place of NAD(H). The schematic representation of the reaction and the kinetics of FgMDH for both forward and the reverse reaction is shown in Fig. 3.

**Table 1:** Kinetic parameters of FgMDH.

Substrate	Co-factor	*K*_m_ (mM)	*V*_max_ (μmol/min)	*V*_max_/*K*_m_
Malate	NAD^+^	0.447 ± 0.047	5.45 ± 0.21	0.0122
Oxaloacetate	NADH	0.276 ± 0.092	4.10 ± 1.10	0.0149
NAD^+^	Malate	0.309 ± 0.015	18.70 ± 0.32	0.0605
NADH	Oxaloacetate	0.141 ± 0.054	3.42 ± 0.87	0.0242

**Table 2:** Comparison of *K*_m_ value of FgMDH with other organisms.

Organism	*K*_m_ malate (mM)	*K*_m_ oxaloacetate (mM)	Ref
*Fasciola gigantica*	0.447 ± 0.047	0.276 ± 0.092	Present study
*Clonorchis sinensis*	0.056 ± 0.006	0.010 ± 0.001	^34^
*Taenia solium*	0.1	0.044	^35^
*Phytophthora infestans*	1.720 ± 0.006	0.176 ± 0.012	^36^

**Figure 4:**
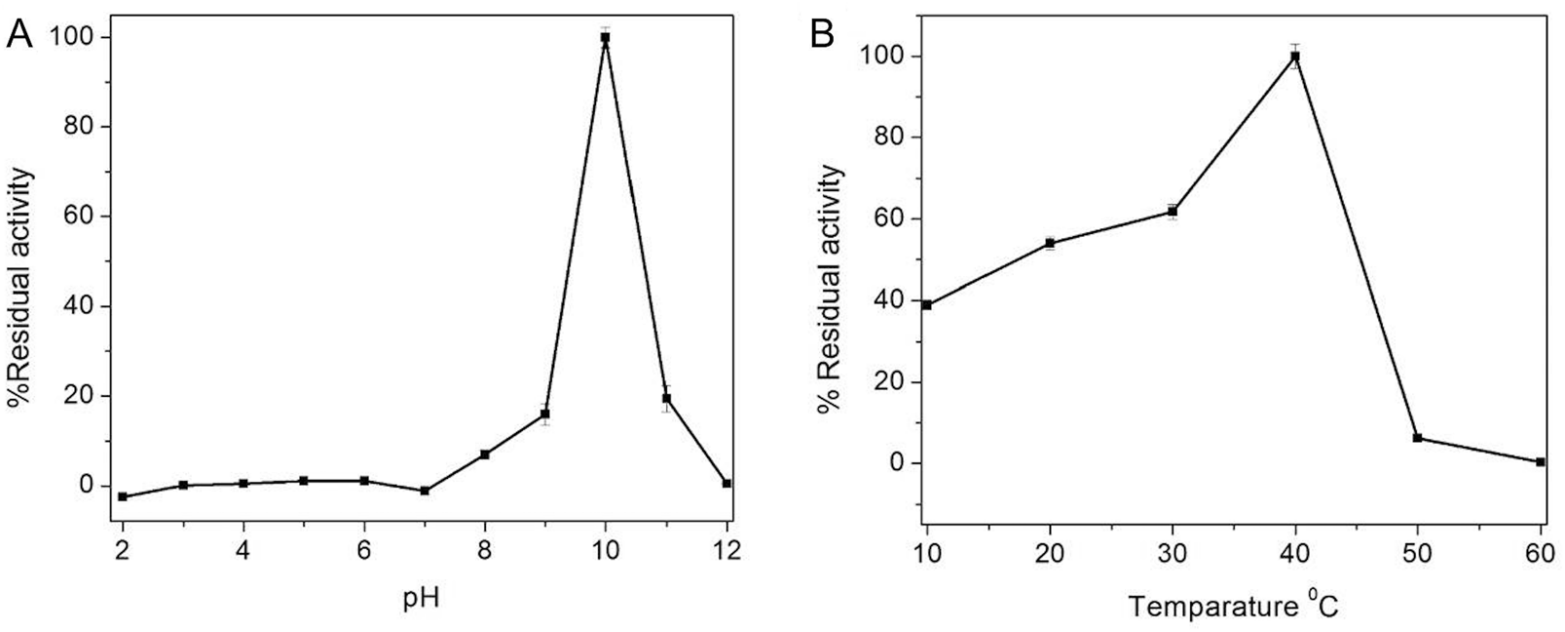
pH and temperature dependent catalytic activity of FgMDH. (**A**) Effect of pH on the catalytic activity of FgMDH. (**B**) Effect of temperature on the catalytic activity of FgMDH.

From the pH-dependent activity of FgMDH, it is evident that the maximum activity was observed at nearly pH 10 in the direction of malate to oxaloacetate. At a pH of less than 7 and more than 10, there is an almost complete loss of enzymatic activity (Fig. 4A). Similarly, in the case of temperature-dependent activity, the activity of FgMDH increased until 40 °C, above which the activity of FgMDH decreased drastically (Fig. 4B).

### Secondary and tertiary structural features

**Figure 5:**
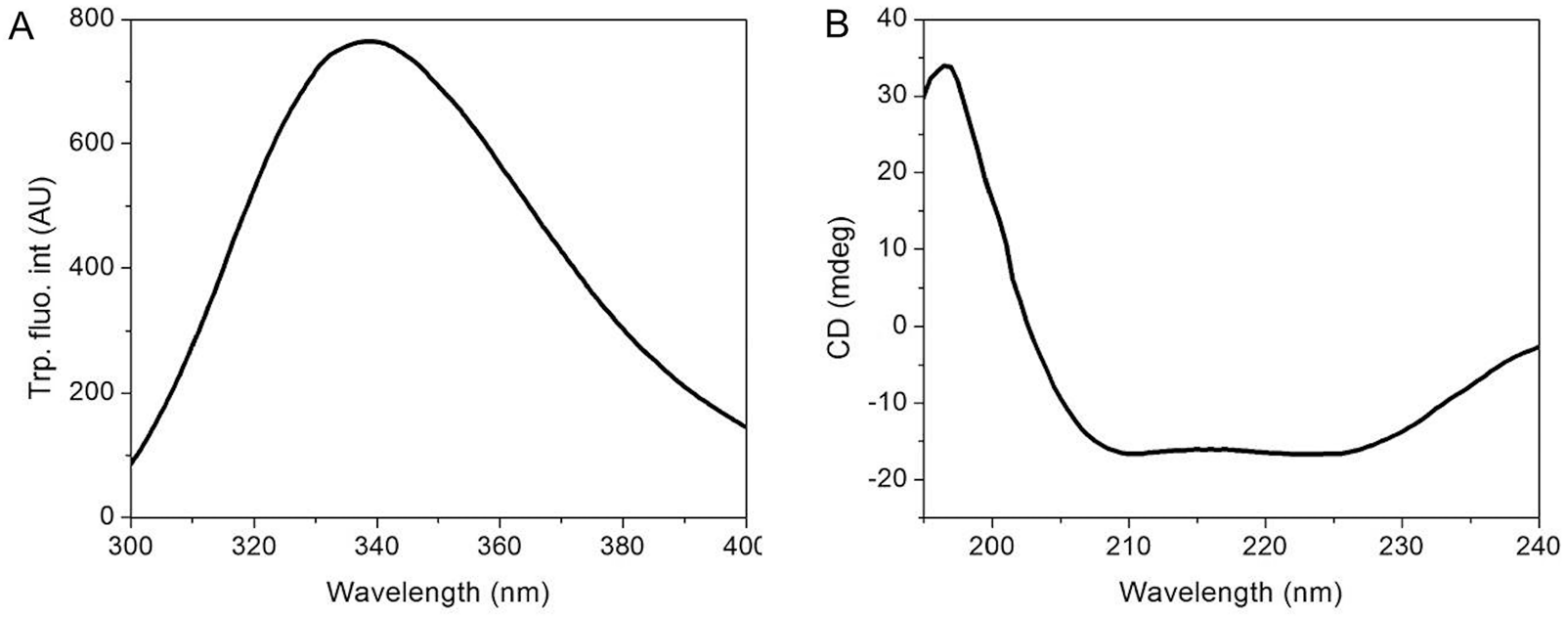
Structural features of recombinant FgMDH. (**A**) Intrinsic tryptophan fluorescence spectrum. (**B**) Far-UV CD spectrum.

To determine the tertiary structure of FgMDH, intrinsic Trp fluorescence was used. The amino acid sequence of FgMDH contained nine Trp residues in each monomer. In the native state, FgMDH showed an emission maximum at nearly 337 nm (Fig. 5A). It is known that when a Trp residue is buried inside the folded protein, it shows a fluorescence emission maximum at 330–335 nm, which signifies that in the case of FgMDH, most of the Trp residues are oriented outward and partially exposed to the solution. To predict the secondary structure of FgMDH, far-UV circular dichroism (far-UV CD) spectroscopy was used. Reportedly, proteins containing α-helices and β-sheets show a characteristic far-UV CD spectrum with two minima at 222 nm and 208 nm for the protein containing α-helices and single minima at 216 nm for the protein containing β-sheets. From the far-UV CD spectrum, it was demonstrated that the secondary structure of FgMDH contains both α-helices and β- sheets (Fig. 5B). The secondary structure of FgMDH and human MDH (HsMDH) were also predicted by using the PDBsum server. The secondary structures of both FgMDH and HsMDH contain 13 α-helices, 14 β-sheets, and 25 coiled conformations (Supplementary Fig. 2A and B).

### Homology modeling and structure validation

From the BLASTp search against the PDB database, it was found that both sequences had high similarity with the *Sus scrofa* MDH. FgMDH showed 99% query coverage and 60% amino acid identity in pairwise alignment, while HsMDH showed 99% query coverage and 95% amino acid similarity in pairwise alignment with SsMDH. Thus, we used the structure of NAD^+^-bound SsMDH (PDB ID: 5MDH, X-ray: 2.4 Å) for the modeling of both the proteins. Eight models were predicted by MODELLER 9.16 for both the proteins and the best models were selected based on DOPE scores (Supplementary Fig. 3A,B). To know the accuracy of the FgMDH and HsMDH models, they were aligned with SsMDH. The root mean square deviation (RMSD) value of 0.14 Å and 0.136 Å was observed for FgMDH (323 C_α_ atoms) and HsMDH (333 C_α_ atoms) respectively, suggesting the model is structurally reliable. Ramachandran plot of the FgMDH model showed 92.8%, 6.2%, 0.7%, and 0.3% residues in most favored regions, additionally allowed regions, generously allowed regions, and disallowed regions, respectively (Supplementary Fig. 4A). While for the HsMDH model, the Ramachandran plot revealed 93.9%, 5.4%, 0.3%, and 0.3% residues in most favored regions, additionally allowed regions, generously allowed regions, and disallowed regions, respectively (Supplementary Fig. 4B). The Z-scores were found to be − 9.92 and − 10.33 for the predicted FgMDH and HsMDH models (Supplementary Fig. 5), whereas it was − 10.19 for template SsMDH structure. The energy plots of FgMDH and HsMDH show all the residues with negative values indicating that the both predicted structure is in stable conformation (Supplementary Fig. 6). Verify3D score was showed to be 84.71 and 88.02 for FgMDH and HsMDH, respectively (Supplementary Fig. 7). All the data suggested that our modeled structures are accurate and reliable. Thus, the modeled structures can be considered on the basis of stereochemical parameters for further structural studies.

**Figure 6:**
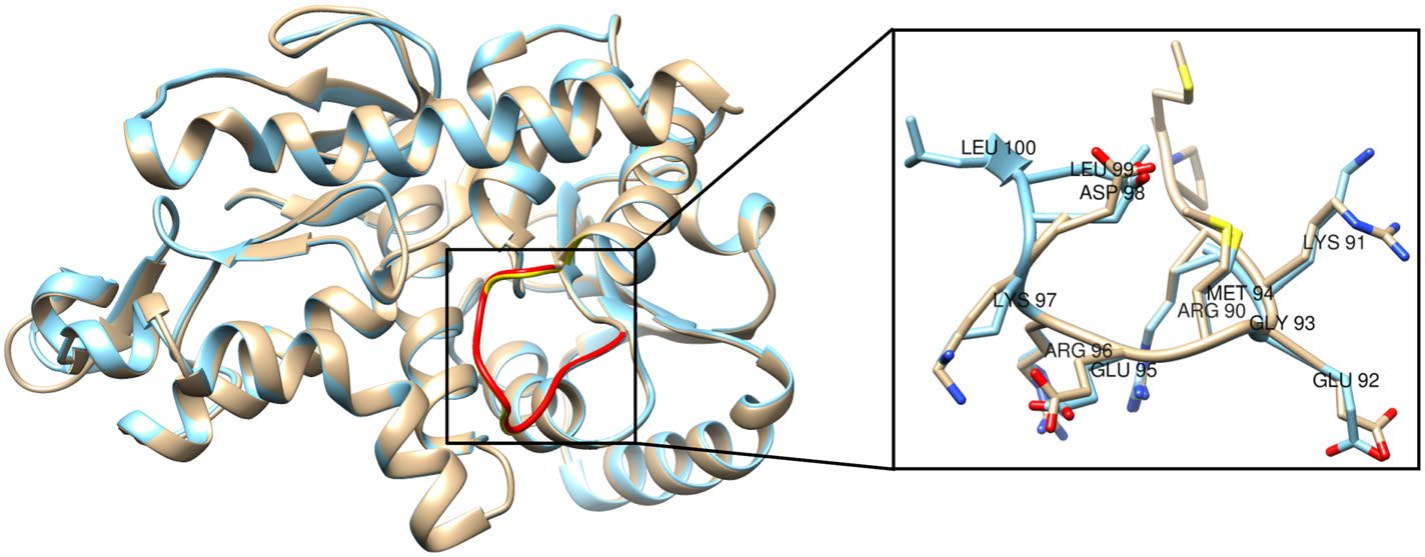
Structural comparison of FgMDH and HsMDH. Structural alignment of FgMDH (cyan) with HsMDH (tan) generates an RMSD of 0.241 Å for 326 C_α_ atoms. High RMSD value indicates lower structural similarity. The inset shows the comparison of the conformation of the active site loop region of FgMDH and HsMDH.

**Figure 7:**
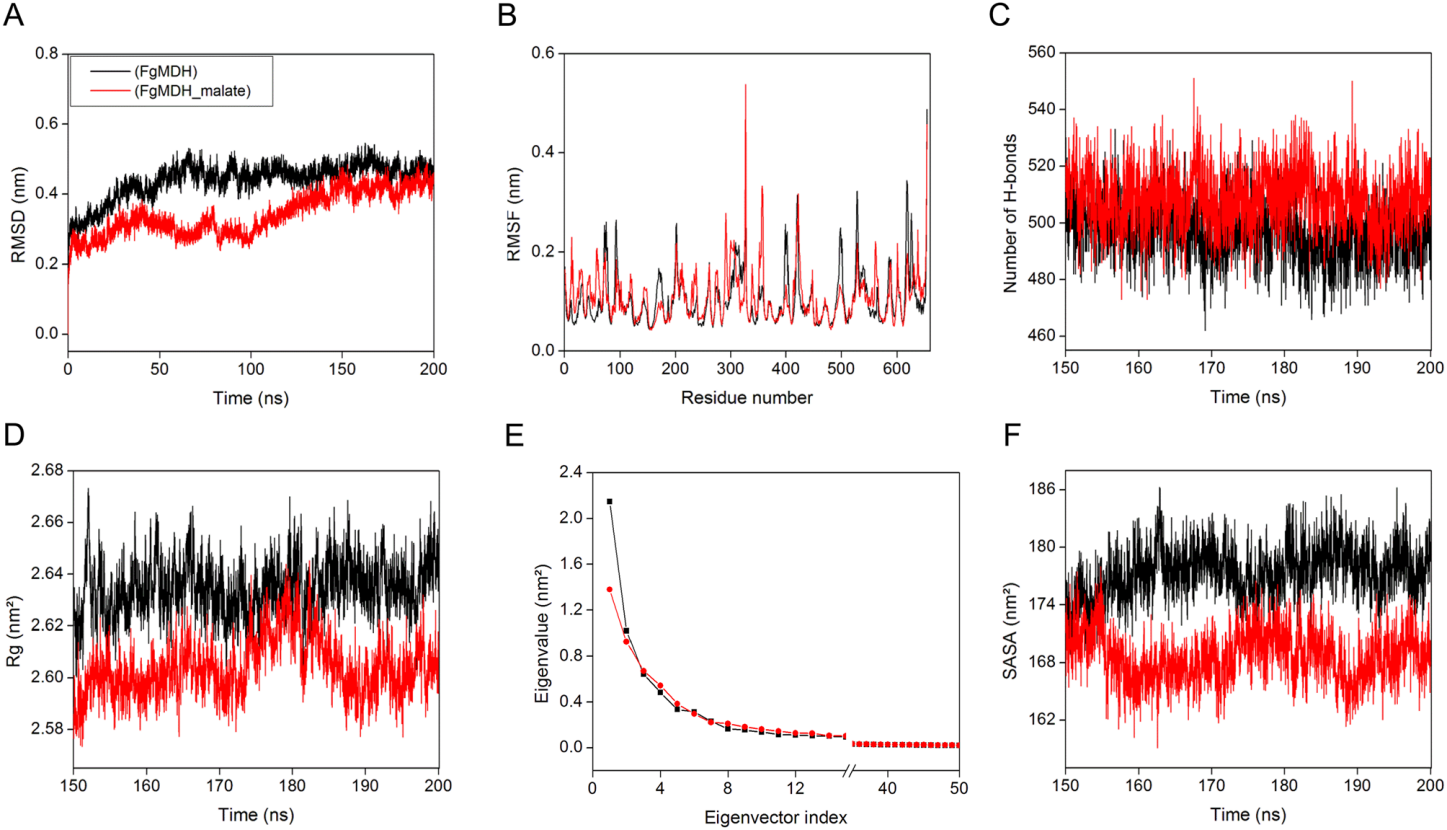
Molecular dynamics simulations analyses of FgMDH and FgMDH–malate complex. (**A**) RMSD of the backbone C_α_ atoms. (**B**) RMSF of C_α_ atoms of last 50 ns MD trajectories. (**C**) Number of hydrogen bonds for the last 50 ns time period. (**D**) The radius of gyration *vs.* time. (**E**) The first 50 PCs *vs.* eigenvectors are shown. (**F**) Solvent accessible surface area.

### Structural comparison of FgMDH with HsMDH

The FgMDH and HsMDH showed ~ 57% sequence identity. To evaluate the structural differences between both proteins, the structures were superimposed. The data showed a minor deviation in the active site loop region and other substrate-binding residues. The substrate binds to the multiple residues of the active site loop region, which is catalytically essential. The superimposed structures showed a similar overall fold and active site loop; however, the side-chain conformation of the loop residues was different (Fig. 6), suggesting structural alterations in the orientation of the active site loop region between these two proteins. The backbone RMSD of the catalytic loop was small (RMSD, 0.09 Å), as the backbone had a similar structure in both the proteins. The overall backbone RMSD of protein superposition was 0.241 Å for 326 C_α_ atom pairs. The cofactor and substrate binding residues are conserved in various MDHs, as shown in Fig. 1A. Thus, FgMDH showed overall structural similarity with the HsMDH, while presenting differences in the conformation of the active site loop residues.

### Molecular dynamics simulations

Molecular dynamics (MD) simulations were performed to study the dynamic properties of the predicted modeled structures of FgMDH and FgMDH–malate complex. All frames were clustered to generate one stable FgMDH structure for substrate docking. Backbone RMSD was calculated from the initial to final trajectories for both the systems. The stability of the protein structure is inversely proportional to the extent of the deviations. The FgMDH and FgMDH–malate complex structures showed an average RMSD value of 0.44 and 0.34 nm until the end of the simulation. Initially, the RMSD values were inconsistent but gained stable trajectories after 50 ns and were stable until the end of the simulation (Fig. 7A). Hence, all further analyses were performed using the average data of the last 50 ns of trajectories. The lower value of RMSD for FgMDH–malate complex suggested increased stability of the complex. The root mean square fluctuations (RMSF) for C_α_ atoms were calculated and plotted, as shown in Fig. 7B. Analysis of the RMSF plot provided information on the flexible regions of the protein. Highly flexible peaks indicate the presence of loops and turns, whereas low values of RMSF indicate rigid regions, such as α-helices and β-sheets. The FgMDH and FgMDH–malate complex showed almost similar fluctuations (average RMSF of 0.10 nm for both systems). The loop regions that ranged from 89 to 109 and 402 to 421 showed higher RMSF values for both systems. Hydrogen bonds play an important role in protein folding and stability, and a higher number of hydrogen bonds indicate a well stable structure. FgMDH and FgMDH–malate complex showed an average of 497 and 508 intermolecular hydrogen bonds during the last 50 ns trajectories (Fig. 7C), indicating that the FgMDH–malate complex formed more number of hydrogen bonds than the FgMDH. The radius of gyration (Rg) is the measure of the compactness of the protein structure^41^. The average Rg value of FgMDH and FgMDH–malate complex were 2.63 nm and 2.60 nm, respectively (Fig. 7D), suggesting that the FgMDH–malate complex was slightly compact than the FgMDH. The correlated motions of FgMDH and FgMDH–malate complex structure was then predicted using the principal component analysis (PCA) method (Fig. 7E). In general, the first few eigenvectors are important for overall correlated motions^42,43^. In FgMDH and FgMDH–malate complex, the first 10 eigenvectors showed 80.74% and 78.30% of motions for the last 50 ns trajectories, respectively. For a better representation of the results, we selected only the first two eigenvectors and created a 2D principal components (PCs) plot, which shows that both FgMDH and FgMDH–malate complex (Supplementary Fig. 8A) form a stable cluster. The solvent accessible surface area (SASA) defines the solvent accessibility of protein; our result shows the average SASA of FgMDH and FgMDH–malate complex were 177 and 168 nm^2^, respectively (Fig. 7F). The average residue SASA of FgMDH and FgMDH–malate complex were 0.493 and 0.474 nm^2^, respectively (Supplementary Fig. 8B). All these data reveal minor compaction and subsequent stabilization in the protein structure upon the formation of the complex with malate. In secondary structure analysis, it was observed that during the MD simulation of FgMDH–malate complex, more number of α-helices and β-sheets were evolving with time, suggesting stability of the system. During the end of the simulation, β-bridges, coils, and bends confirmations were lesser, whereas the number of α-helices and β-sheets were more in FgMDH–malate complex (Supplementary Fig. 9) than in FgMDH than the initial structure.

**Figure 8:**
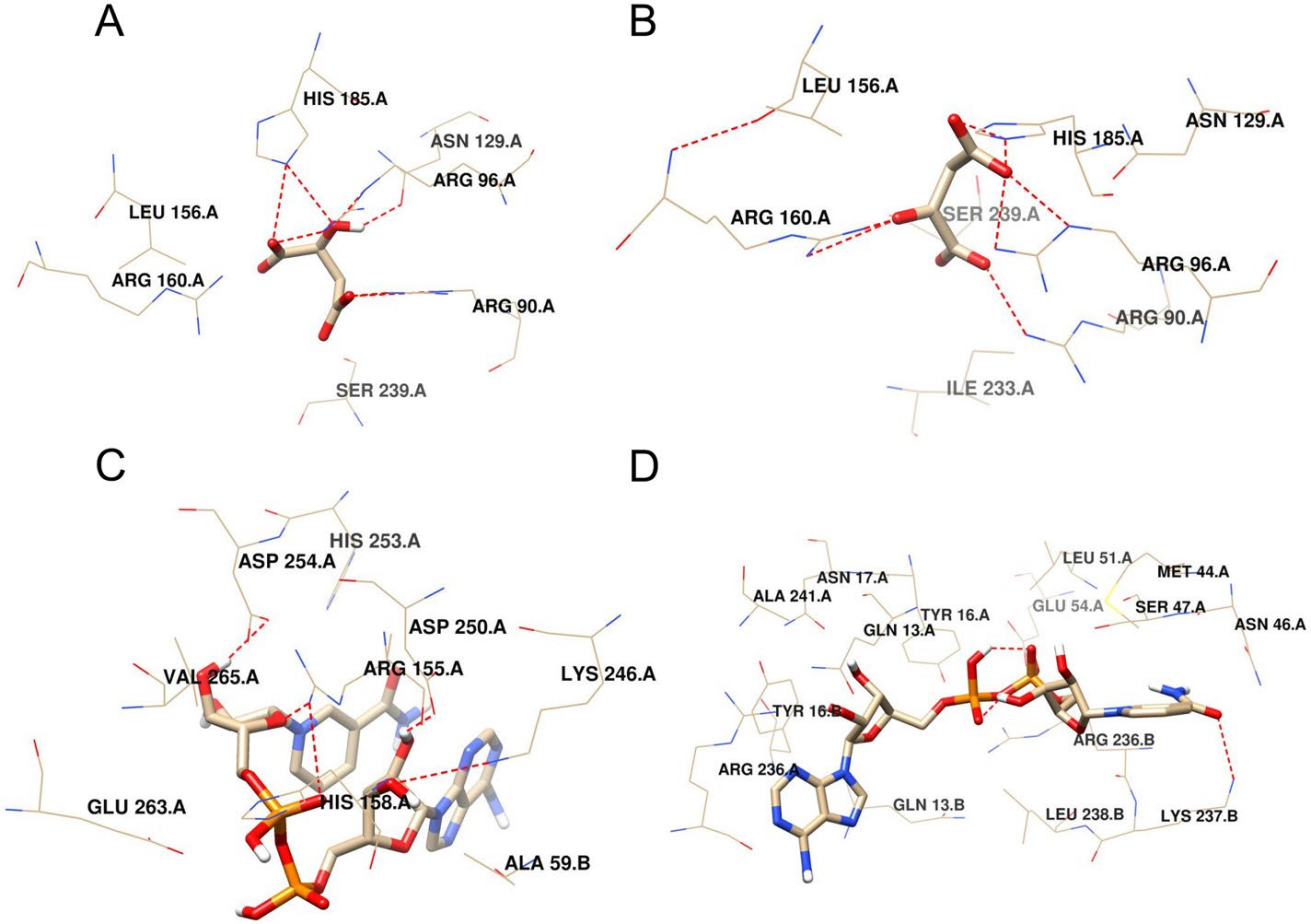
Interaction of substrates and cofactors with FgMDH. The dotted line represents the H-bond with (**A**) Malate, (**B**) Oxaloacetate, (**C**) NAD^+^, and (**D**) NADH.

### Molecular docking

The structure of SsMDH (PDB ID: 5MDH) was an NAD^+^-bound complex. The active site residues (both substrate and cofactor binding site) in SsMDH were Arg91, Arg97, Asp158, His186, Arg161, Ser241, Gly13, Ile15, Asp41, and Ser240 corresponding to Arg90, Arg96, Asp157, His185, Arg160, Ser240, Gly13, Ile14, Asp40, and Ser239 in FgMDH, respectively. Thus, we selected these residues for further molecular docking with the average stabilized structure. The lowest binding energy was selected with the top pose from docking. The best pose that formed the low energy complex with NAD^+^ and NADH showed binding energy of − 9.1 kcal·mol^−1^, whereas the complex with malate and oxaloacetate showed binding energy of − 5.2 kcal·mol^−1^ (Table 3). The substrates (malate and oxaloacetate) and cofactors (NAD^+^ and NADH) interacting residues are shown in Fig. 8 and Table 3. Malate interacted with only one chain with residues Arg90, Ser240, and Asn129 (one hydrogen bond each) and with Arg96 and His185 (two hydrogen bonds each). Oxaloacetate also interacted with only one chain and formed one hydrogen bond with Arg90, Ser240, and His185, and two hydrogen bonds with Arg96 and Arg160. FgMDH formed nine hydrogen bonds, each with both NAD^+^ and NADH, whereas seven hydrogen bonds each with malate and oxaloacetate. The residues that formed hydrogen bonds with NAD^+^ in chain A were Asp254 and His158 (one hydrogen bond each), whereas in chain B were Arg24 (one hydrogen bond), Arg155, Lys246, and Asp250 (two hydrogen bonds each). NADH formed hydrogen bonds with Asn46, Ser47, and Glu54 (one hydrogen bond each) and with Arg236 in chain A (two hydrogen bonds), whereas in chain B were Ala11, Ala235, Arg236, and Lys237 (one hydrogen bond each).

**Table 3:** Summary of docking energy, interacting residues and atoms involved in H-bonding with substrates and cofactors. Data obtained using molecular docking studies by Autodock Vina.

Substrate/co-factor	Average binding energy (kcal·mol^−1^)	No of H-bonds residues	Interacting residues
**Malate with FgMDH**	−5.2	A:Arg90:NH2—Lig:O5 A:Arg96:NH2—Lig:O1 A:Arg96:NH2—Lig:O2 A:Ser240:N—Lig:O4 Lig:H13—A:Asn129:OD1 A:His185:NE2- Lig:O1 A:His185:NE2- Lig:O2	Chain A Ser239, Leu156 ,Arg160
**Oxaloacetate with FgMDH**	−5.2	A: Arg 90: NH2—Lig: O5 A: Arg 96: NE—Lig: O2 A: Arg 96 NH2—Lig: O2 A :Arg 160: NH1—Lig: O1 A :Arg 160: NH2—Lig: O1 A:His 185: NE2—Lig:O2 A:Ser 240: N—Lig:O4	Chain A Leu156, Asn129, Ser239, Ile233
**NAD**^**+**^** with FgMDH**	−9.1	A: Arg 155: NH2—Lig: O15 A: Arg 155 NH2—Lig: O4 A:Lys 246: NZ—Lig: O5 A:Lys 246: NZ – Lig:O6 Lig:N22—B: Arg 24: O Lig: O13—A:His 158: NE2 Lig: O6—A: Asp 250: OD1 Lig: O6—A:Asp 250: OD2 Lig: O7—A:Asp 254: OD1	Chain A Val265, His253, Glu263 Chain B Ala59
**NADH with FgMDH**	−9.1	A:Asn46:ND2—Lig:O16 A:Ser47:OG—Lig:O15 A:Arg236:NH2—Lig:O6 A:Arg236:NH2—Lig:N20 B:Arg236:NH2—Lig:O11 B:Lys237:NZ—Lig:O16 Lig:N23—B:Ala235:O Lig:N22—B:Ala11:O Lig: O13 A:Glu 54: OE2	Chain A Leu51, Met44, Ala241, Asn17, Tyr16, Gln13 Chain B Gln13, Tyr16, Leu236

## Conclusions

The recent availability of the *F. gigantica* genome has provided a basis for the biochemical and structural characterization of the proteins of this parasite^36^. It has been reported that in addition to the aerobic metabolism, cMDH also plays a key role in the anaerobic metabolism of the liver flukes. When the liver flukes penetrate the host bile duct, the parasite metabolism shifts from aerobic to anaerobic. The main sources of ATP during anaerobic metabolism in the bile duct are acetate and propionate. In the adult liver flukes, PEPCK catalyzes the conversion of PEP to oxaloacetate in the presence of ATP. The cMDH converts this oxaloacetate into malate with the concomitant production of NAD^+^. The malate is then transported to the mitochondria and degraded through malate dismutation; part of the malate is oxidized to acetate and the other part is reduced to succinate, which is subsequently converted to propionate. We observed that the FgMDH catalyzed both forward and reverse reactions with high efficiency, suggesting the key role of this enzyme in the parasite. The overall structure of the FgMDH was conserved and showed high similarity with the human counterpart; however, it presented differences in the conformation of the active site loop residues. The slight conformational differences in the loop residues could be exploited in the development of targeted, fluke-specific inhibitors. This study of the catalytic and structural properties of *F. gigantica* MDH is crucial in understanding the roles of this evolutionarily conserved enzyme in the parasite. The work also provides new insights into the biochemistry of liver flukes.

## Methods

### Phylogenetic analysis

FgMDH sequence was submitted to the NCBI-BLAST search to predict homologs. A total of 11 homologous sequences were selected and aligned using ClustalW algorithm^44^ (https://www.genome.jp/tools-bin/clustalw) with its default parameters. ESpript 3.0 software was used to generate the color-coded version of multiple sequence alignment. Phylogenetic analysis and tree construction were performed by MEGA V10.0 software^45^.

### Parasite collection

Adult liver flukes were collected from naturally infected animals from Bada Bazaar abattoir, Shillong, India. The flukes were thoroughly washed with chilled 1 × phosphate-buffered saline (PBS, pH 7.4), frozen in liquid nitrogen, and stored at − 80 °C until use.

### RNA isolation and reverse transcription for cDNA synthesis

Total RNA was isolated using RNAse easy mini kit (Qiagen). cDNA was synthesized using mRNA as a template and QuantiTect reverse transcription kit (Qiagen).

### Gene amplification and cloning of *Fgmdh*

The gene amplification of full-length *Fgmdh* (981 bp) was performed in a thermocycler from cDNA using Phusion High-Fidelity DNA Polymerase and gene-specific primer pairs with different restriction sites. The forward and reverse primer sequences were 5′-GGATCCATGGCTCCTGTCAAAGTGCTC-3′ and 5′-AAGCTTTTTTCCAAGCGCAGCAACCTT-3′ with BamHI and HindIII restriction sites (underlined), respectively. The other components of PCR include HF buffer, dNTPs and deionized water prepare the complete reaction mixture. PCR program was performed as follows: initial denaturation at 98 °C for 30 s followed by 33 cycles of amplification consisting of denaturation at 98 °C for 10 s, annealing at 58 °C for 10 s, extension at 72 °C for 25 s, and a final extension at 72 °C for 5 min. The amplified product was separated using 1% agarose gel electrophoresis in 1 × tris–acetate-EDTA buffer. DNA band was extracted, and the gel was purified using the QIAquick Gel Extraction Kit (Qiagen). The amplified gene was then cloned into cloning vector pSK^+^ and then transformed into the *E. coli* DH5α cells. The transformed product was spread onto the agar plate containing ampicillin (100 µg/mL) with X-gal and β-d-1-thiogalactopyranoside (IPTG) for the blue-white screening of positive clones. The positive clones were also screened by restriction digestion and further confirmed by sequencing. The clone without any mutation in the *mdh* gene was selected and sub-cloned into the pET28a( +) expression vector at the defined restriction sites.

### Overexpression and purification of FgMDH

The recombinant FgMDH-pET28a( +) construct was transformed into *E. coli* codon( +) expression cells and plated into the agar plate containing kanamycin (50 µg/mL). A single colony was picked and inoculated into a 5 mL culture vial containing 50 µg/mL of kanamycin and grown overnight at 37 °C with continuous shaking at 180 rpm. This primary culture was inoculated in 500 mL of Luria–Bertani (LB) media containing 50 µg/mL kanamycin and grown at 37 °C with continuous shaking until the OD_600_ reaches ~ 0.6–0.8. The culture was then cooled down and induced with 1.0 mM IPTG and incubated at 20 °C with shaking at 150 rpm for 24 h. The culture was pelleted by centrifugation at 4 °C, dissolved in 30 mL of buffer A (50 mM phosphate, pH 7.6, containing 150 mM NaCl), and 10% glycerol (v/v), and stored at − 80 °C until further use. Before sonication, 1 mM phenylmethylsulfonyl fluoride (PMSF) was added, and the cells were disrupted in ice (50 Hz amplitude, 30 s pulse with 30 s interval, 30 cycles). The cell lysate was centrifuged at 12,000 rpm for 30 min at 4 °C. The supernatant was filtered through a 0.45-μm pore size polyvinylidenedifluoride membrane before loading onto the Ni^2+^–NTA affinity matrix. The beads were washed with a 10-bed volume of buffer A (equilibration buffer). The supernatant was applied to the Ni^2+^–NTA column, followed by washing with an increasing concentration of imidazole. The recombinant protein was finally eluted with elution buffer (50 mM phosphate, pH 7.6, containing 150 mM NaCl, 10% glycerol, and 300 mM imidazole). The concentration of the purified protein was measured spectrophotometrically by the Bradford method using bovine serum albumin as a standard, and purity of the protein was analyzed with 12% SDS-PAGE. The protein was dialyzed overnight at 4 °C in dialysis buffer (buffer B, 50 mM phosphate pH 7.6, containing 150 mM NaCl, and 10% glycerol).

### Determination of the oligomeric status of recombinant FgMDH

The oligomeric status of recombinant FgMDH protein was determined by GFC on a Superdex™ 200 column on an ÄKTA fast protein liquid chromatography instrument. The column was equilibrated with buffer B at room temperature; 500 μL of affinity-purified FgMDH was loaded onto the column and eluted at a flow rate of 0.3 mL/min. The protein peak was detected at 280 nm.

### Enzymatic activity of FgMDH

The enzymatic activity of FgMDH was measured spectrophotometrically for the oxidation and reduction of NADH at 340 nm. For the oxaloacetate reduction assay, 1 mL reaction mixture contained 50 nM FgMDH, 0.21 mM NADH, and 50 mM Tris–Cl at 25 °C and pH 8.0. The reaction was initiated by adding 0.2 mM oxaloacetate and, the absorbance was recorded at 10 s intervals for 60 s. Similarly, for the oxidation of MDH, 1 mL of assay mixture contained 50 nM FgMDH, 0.5 mM NAD^+^, and 50 mM glycine buffer at 25 °C and pH 9.6^46^. The reaction was initiated by adding 3 mM of MDH, and the absorbance was recorded at 10 s intervals for 60 s. The kinetic parameters for both substrate and cofactor were determined by varying the concentration of one while keeping other constant. Michaelis–Menten graph was used to fit the data using non-linear regression.

### Temperature and pH-dependent activity of FgMDH

The enzyme activity of FgMDH at different pH was determined by incubating the protein in 50 mM citrate–glycine–hepes buffer containing 150 mM of NaCl at pH 2–12. The reaction was initiated as described earlier, and the residual activity was plotted against different pH range. Similarly, activity at different temperatures was determined by incubating the protein at various temperatures ranging from 10 to 90 °C. All the experiments were repeated in triplicate, and the mean value of all was considered.

### Structural analysis of FgMDH using fluorescence and Far-UV CD spectroscopy

To study the tertiary structure of FgMDH, the Trp fluorescence emission spectrum was recorded at 25 °C in a 10 mm quartz cuvette. The excitation wavelength was kept as 280 nm, and the emission spectrum was recorded between 300 and 500 nm. Far-UV CD spectrum was measured in a 1 mm quartz cuvette. 2 μM protein concentration was used, and the CD spectrum was collected at a scan speed of 50 nm/min, with a response time of 1 s and a bandwidth 2 nm. All the experiments were performed in triplicates, and the background data were subtracted in all the experiments.

### Homology modeling and structure validation

The FgMDH and HsMDH sequence were submitted to PDB–BLAST to predict closely related homologs. Structure of *S. scrofa* MDH (PDB ID: 5MDH) was selected as a template for homology modeling for both the proteins. The sequences were aligned using Clustal W and color-coded alignment was generated by using ESpript3.0 server^47^. The structures were modeled based on the crystallographic information of SsMDH using MODELLER9.16^48^. The predicted models were validated by structure alignment using different software and tools, such as Chimera 1.10.2^49^, PROSA^50^, PDBsum^51^, and Verify3D^52^ servers.

### Cavity identification and binding site analysis

The modeled FgMDH structure was used to find the binding sites. SsMDH structure co-crystallized with cofactor and substrate analogs (PDB ID: 5MDH) was used to find out the binding site and residues^53^. Chimera1.10.2 was used to visualize various bonding and interactions. In SsMDH, substrate-binding residues included Arg91, Arg97, Asp158, His186, and Ser241, whereas cofactor binding residues included Gln13, Ile15, Asp41, and Ser240. Of these, Arg91 and Arg97 are located in the active site loop region. The FgMDH model was superimposed with SsMDH using Chimera1.10.2^49^. The binding site cavity associated with these residues was selected for molecular docking.

### Molecular docking

The substrates (malate and oxaloacetate) and cofactors (NAD^+^ and NADH) were docked with FgMDH using AutoDock Vina^54^. The structures of FgMDH and ligands were prepared by MGL tools. Hydrogen atoms and Kollman charges were added to the structure of FgMDH. Polar hydrogens were removed from ligands; Gasteiger charges were assigned to substrate and cofactor. A 3D grid box was set for docking into X = 66°, Y = 36°, and Z = 98°, and the spacing of the grid was 0.347 Å for the substrate and cofactors. All other parameters were kept as default. For each of the ligand (the substrates and cofactors), nine poses were generated. The best pose was selected for further analysis.

### MD simulations

200 ns MD simulations were performed using GROMACS 4.6.5 in an *in house* supercomputer as described earlier^55–58^. The systems (FgMDH and FgMDH-malate complex) were solvated using a simple point charge. Protein topologies were produced by using GROMOS 9653a6 force-field^59^. Malate topology was generated using ProDRG server^60^. Since the systems were already found to be neutral, no extra ions were added for neutralization of the system. To discard steric clashes, the steepest energy minimization was performed for both systems to keep the maximum force at less than 1,000 kJ mol nm^−1^. Particle Mesh Ewald method was used for calculating long-range electrostatic interactions, and a 1.6 Å Fourier grid spacing was used; 1.0-nm cut-off was kept for calculating Lennard–Jones and Coulomb interactions. For predicting short-range non bonded interactions, 10 Å cut-off distance was considered. The linear constraint solver algorithm was performed to constrain the lengths of hydrogen bonds^61^. All the bonds were fixed by Shake algorithm^62^. Both systems were equilibrated after energy minimization and performed for 1 ns of NVT and NPT. Finally, both the systems were submitted for 200 ns MDS. The trajectories were analyzed using g_rmsd, g_rmsf, g_gyrate, g_hbond, g_cover, and g_anaeig tools, as described previously^55–58^. The trajectory of the systems was analyzed by visual molecular dynamics^63^ and Chimera. All the graphs were plotted in Origin 6.0 and visualized.

## Supplementary information

Supplementary Information.
