# Supplementary material for: Identification and characterization of cytosolic malate dehydrogenase from the liver fluke Fasciola gigantica
Source: Sci Rep. 2020 Aug 7;10:13372. doi: 10.1038/s41598-020-70202-y (PMC7415141; doi:10.1038/s41598-020-70202-y)

**SUPPLEMENTARY INFORMATION**

**Identification and characterization of cytosolic malate dehydrogenase from the liver fluke *Fasciola gigantica***

Purna Bahadur Chetri, Rohit Shukla^#^ and Timir Tripathi*

Molecular and Structural Biophysics Laboratory, Department of Biochemistry, North-Eastern Hill University, Shillong 793022, India

*Running title: Characterization of F. gigantica MDH*

**^*^Corresponding author**: Dr. Timir Tripathi, Department of Biochemistry, North-Eastern Hill University, Shillong- 793022, India. Email: timir.tripathi@gmail.com. Tel: +91-364-2722141; Fax: +91-364-2550108.

^#^Current address: Department of Biotechnology and Bioinformatics, Jaypee University of Information Technology, Waknaghat, Solan- 173234, India

**Supplementary Fig. S1: PCR amplification and restriction digestion of the FgMDH gene.** Lane 1, DNA ladder. Lane 2: amplified PCR product (981 bp). Lane 3: *mdh*-pSK+ digested with *Bam*HI and *Hind*III; upper band represents the linear backbone of pSK+ vector (~3 kb) and lower band (981 bp) is the *mdh* gene. Lane 4: *mdh*-pET28(a) digested with *Bam*HI and *Hind*III; upper band represents the linear backbone of pET28(a) vector (~5.3kb) and lower band (981bp) is the *mdh* gene. A 1% agarose gel electrophoresis was used to visualize the bands.


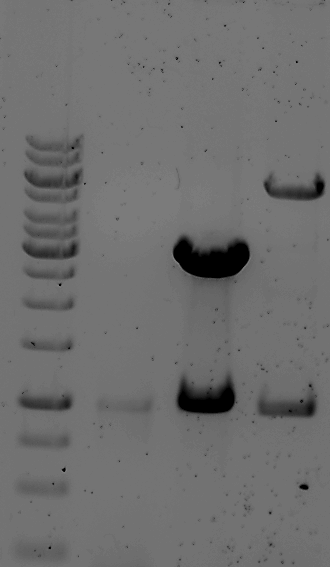


1 2 3 4

5.3 kb

2.9 kb

~1 kb

**Supplementary Fig. S2:** Secondary structure of (A) FgMDH and (B) HsMDH.

**
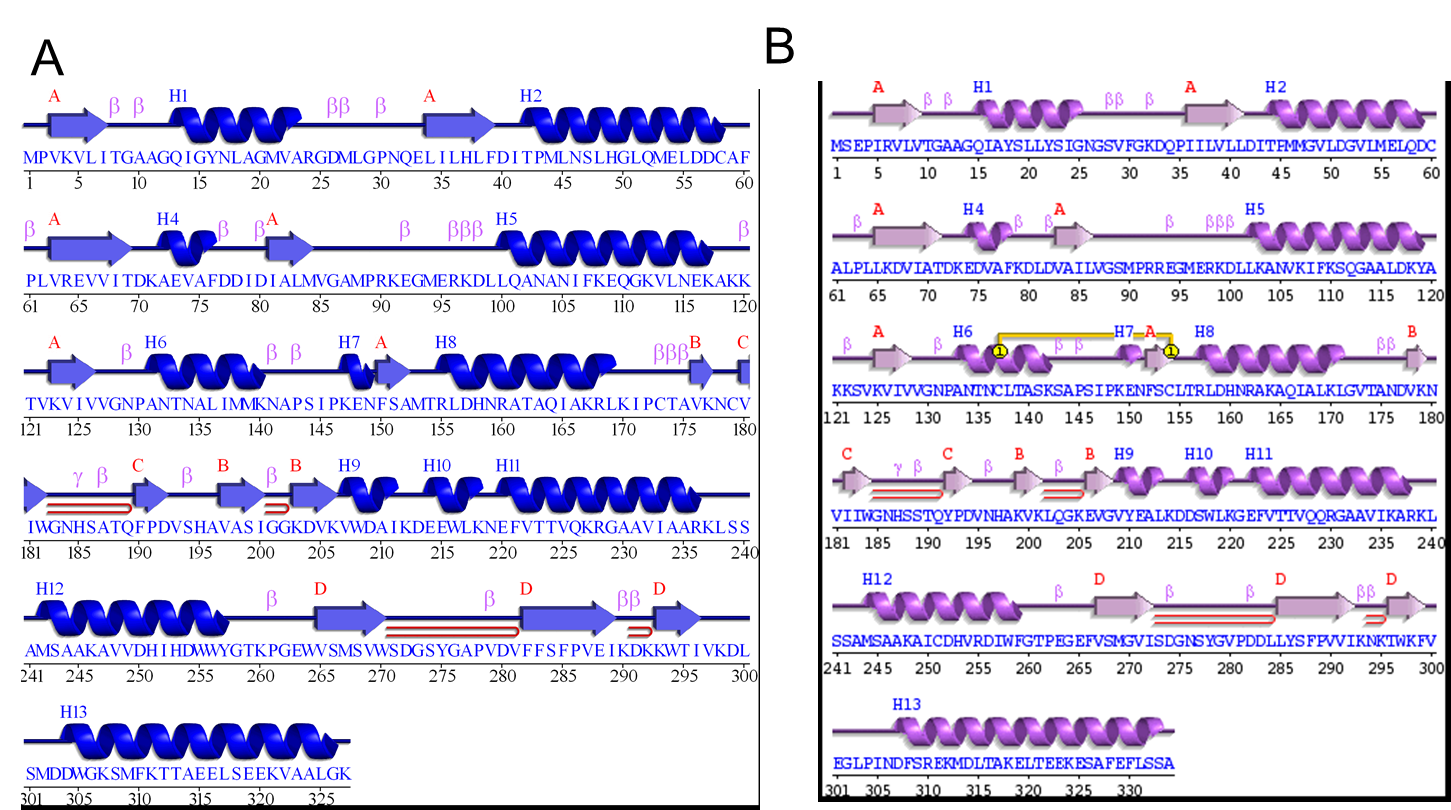
**

**Supplementary Fig. S3:** Modelled structures of (A) FgMDH (B) HsMDH.

**
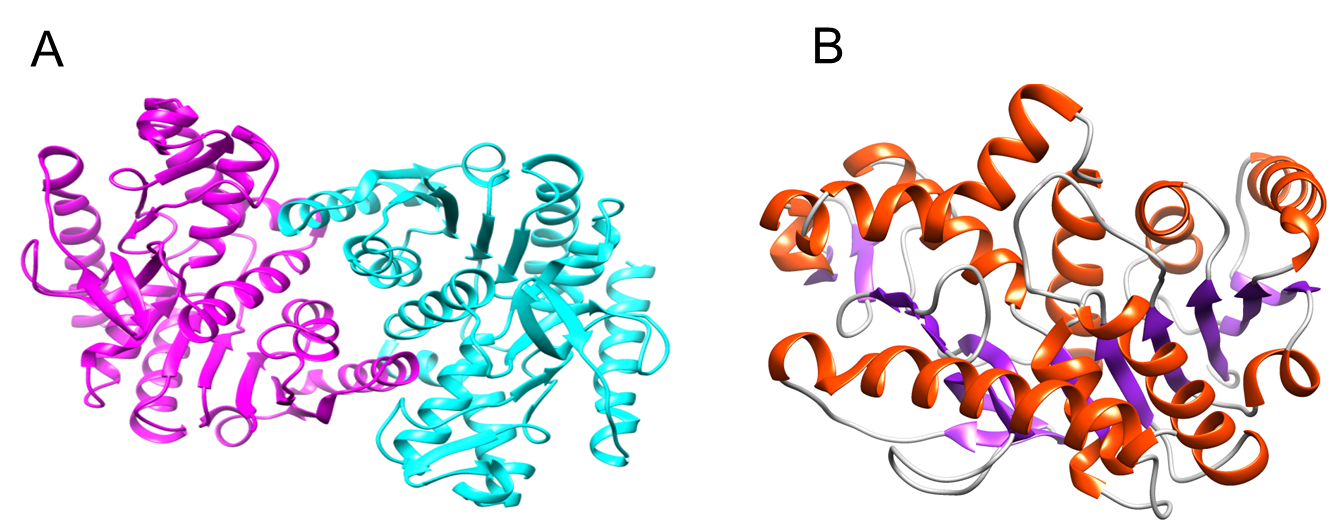
**

**Supplementary Fig.S4:** Ramachandra plot of (A) FgMDH and (B) HsMDH.

**
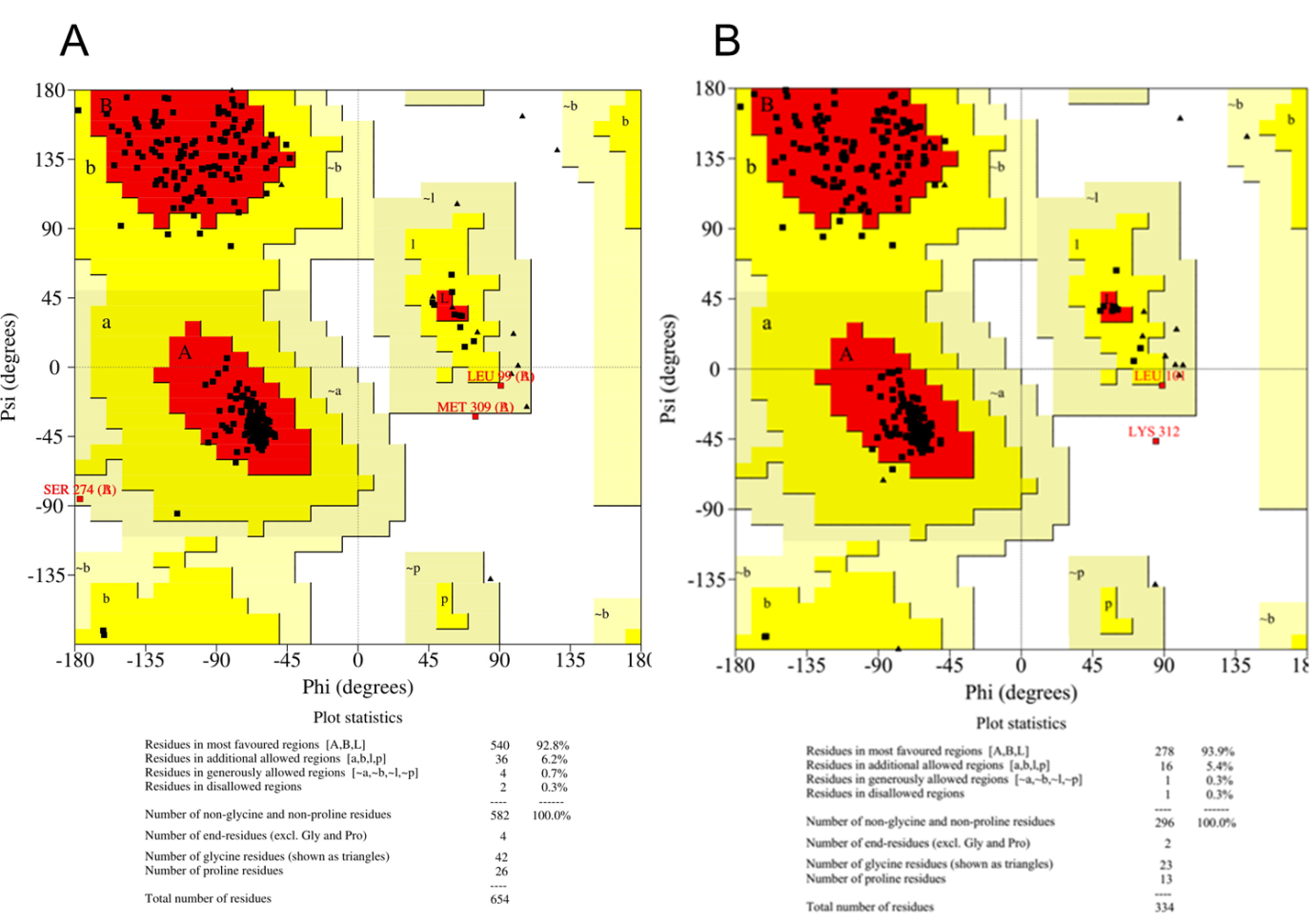
**

**Supplementary Fig. S5:** Z-score plot of (A) FgMDH and (B) HsMDH. The round black color shows the Z-score of the protein FgMDH (-9.92) and HsMDH (-10.33). Z-Score plot depicts z-scores of all experimental protein chains in PDB defined by NMR spectroscopy (dark blue) and X-ray crystallography (light blue).

**
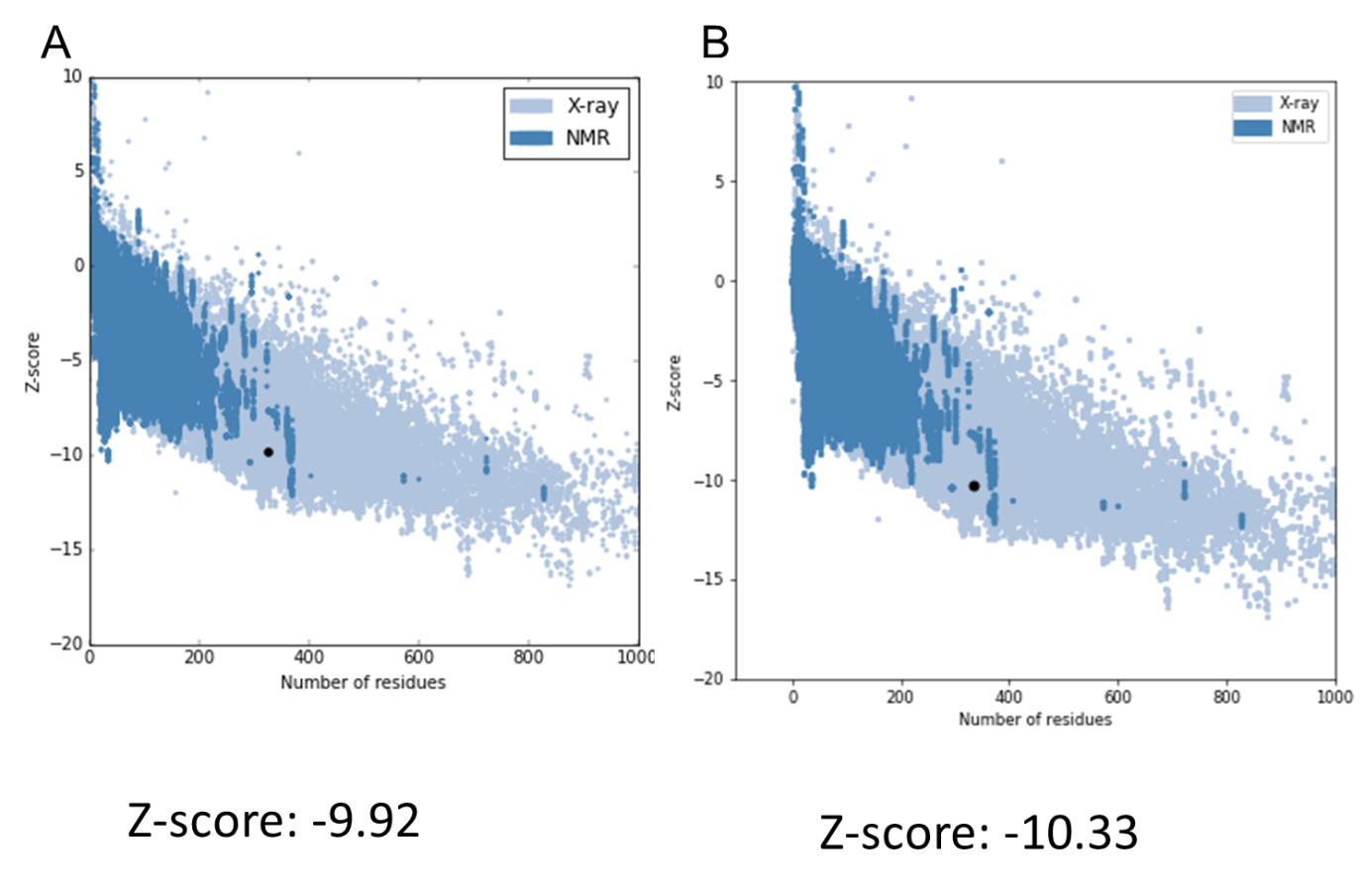
**

**Supplementary Fig.S6:** Energy plot of (A) FgMDH and (B) HsMDH. All the residues fall in the negative window.


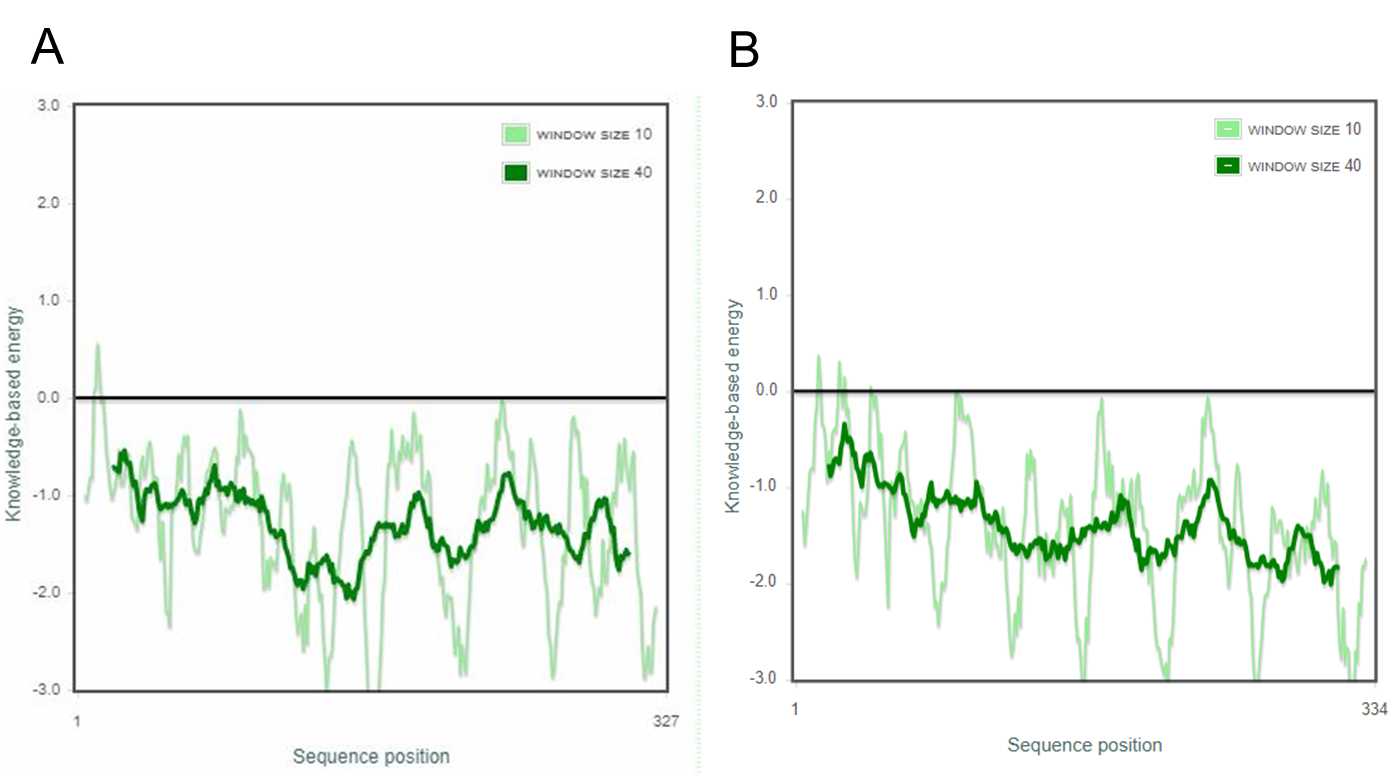


**Supplementary Fig. S7:** Verify-3D plot of (A) FgMDH and (B) HsMDH.

**
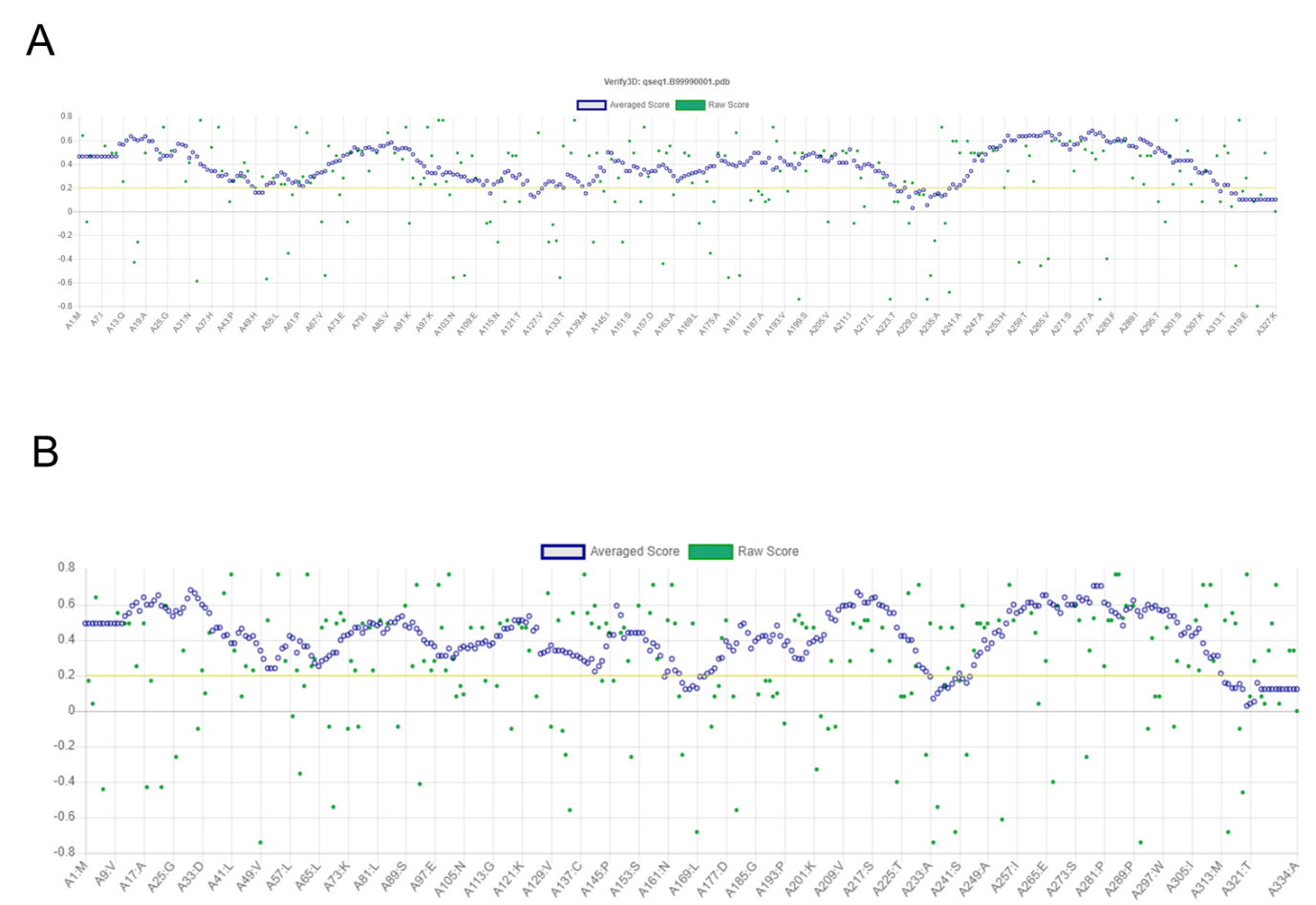
**

**Supplementary Fig. S8:** (A) 2D projection plot for the first two eigenvectors of FgMDH (B) Residue solvent accessible surface area for FgMDH.

**
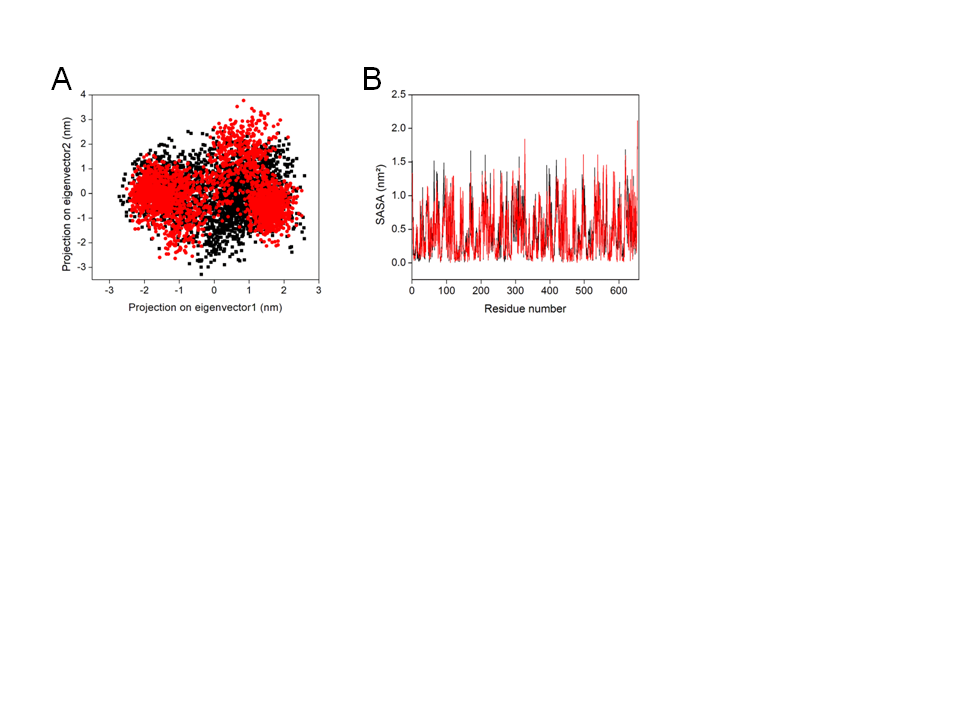
Supplementary Fig. S9:** Secondary structure of (A) FgMDH and (B) FgMDH-malate complex.


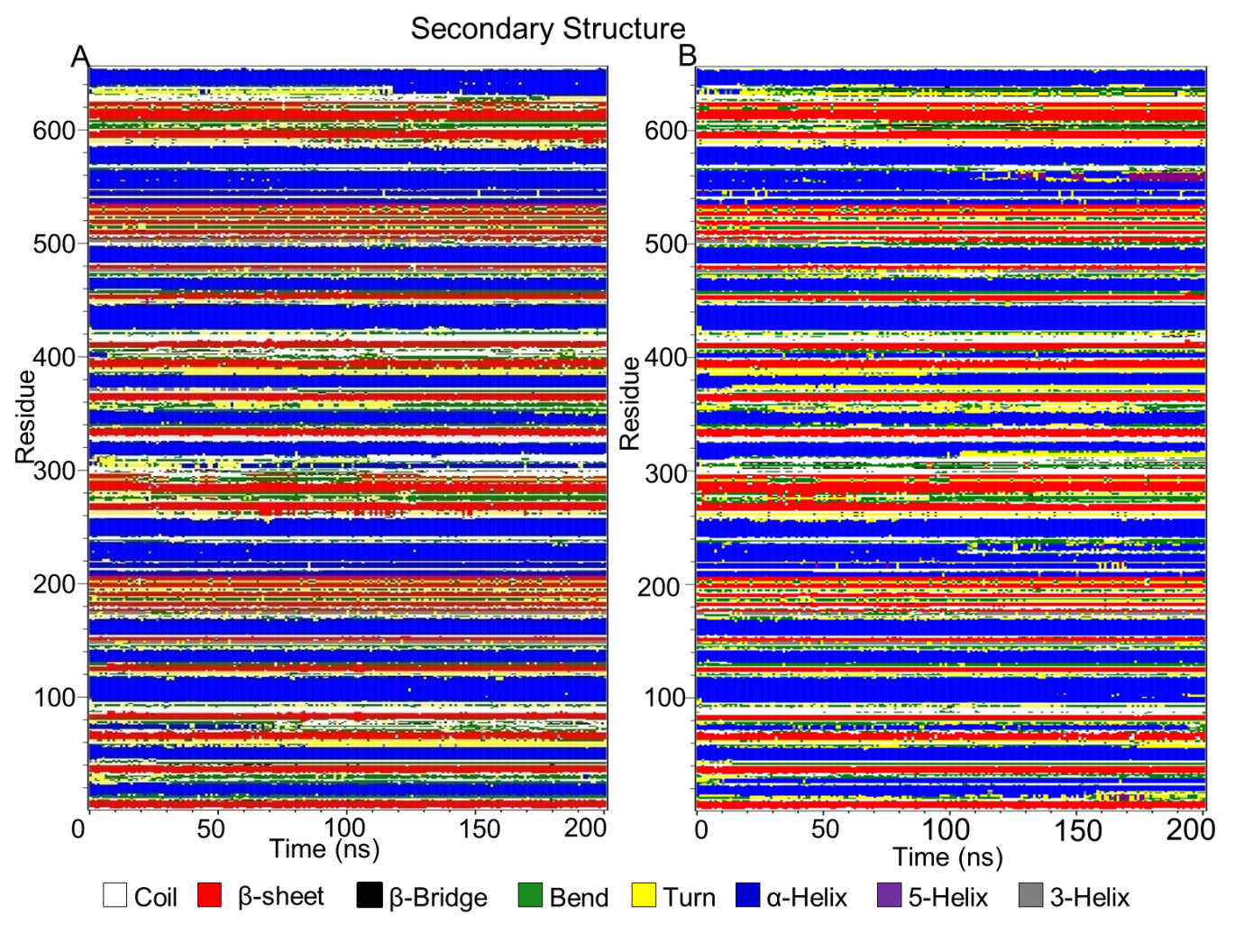

Supplement: Supplementary file 1 — Supplementary Information. [file 41598_2020_70202_MOESM1_ESM.docx]
